# Supplementary material for: The social specificities of hostility toward vaccination against Covid-19 in France
Source: PLoS One. 2022 Jan 6;17(1):e0262192. doi: 10.1371/journal.pone.0262192 (PMC8735622; doi:10.1371/journal.pone.0262192)
Supplement: S1 Table — (DOCX) [file pone.0262192.s001.docx]

|  | **Men** | **Women** | **Total** |
| --- | --- | --- | --- |
| **Total** | 39024 (48) | 46831 (52) | 85855 (100) |
| **Regarding the possibility of contracting the virus in the coming months, would you say that you are afraid of contracting it and being seriously ill?** | | | |
| Yes | 8147 (20,9) | 12021 (27,1) | 20168 (24,1) |
| No | 30877 (79,1) | 34810 (72,9) | 65687 (75,9) |
| **Has taken a Covid-19 test** | | | |
| Yes | 13861 (33,8) | 18688 (37,4) | 32549 (35,7) |
| No | 25027 (65,8) | 27991 (62,1) | 53018 (63,9) |
| Missing | 136 (0,4) | 152 (0,5) | 288 (0,4) |

**S1 Table : Covid-19 test and scare of contracting the virus and being seriously ill, according to sex**
