# Supplementary material for: The social specificities of hostility toward vaccination against Covid-19 in France
Source: PLoS One. 2022 Jan 6;17(1):e0262192. doi: 10.1371/journal.pone.0262192 (PMC8735622; doi:10.1371/journal.pone.0262192)
Supplement: Attachment — Submitted filename: Response to Reviewers.docx [file pone.0262192.s002.docx]

Journal Requirements:

When submitting your revision, we need you to address these additional requirements.

1. Please ensure that your manuscript meets PLOS ONE's style requirements, including those for file naming. The PLOS ONE style templates can be found at

<https://journals.plos.org/plosone/s/file?id=wjVg/PLOSOne_formatting_sample_main_body.pdf> and

<https://journals.plos.org/plosone/s/file?id=ba62/PLOSOne_formatting_sample_title_authors_affiliations.pdf>

***The manuscript was modified so as to meet PLOS ONE’s style requirements.***

2. Thank you for stating the following financial disclosure:

“NB has received funding from the European Research Council (ERC) under the European Union’s Horizon 2020 research and innovation programme (grant agreement No. [856478])”

Please state what role the funders took in the study. If the funders had no role, please state: "The funders had no role in study design, data collection and analysis, decision to publish, or preparation of the manuscript."

If this statement is not correct you must amend it as needed.

Please include this amended Role of Funder statement in your cover letter; we will change the online submission form on your behalf.

***"The funders had no role in study design, data collection and analysis, decision to publish, or preparation of the manuscript." added to the text in the “Funding details” statement and to the Cover letter.***

3. In your Data Availability statement, you have not specified where the minimal data set underlying the results described in your manuscript can be found. PLOS defines a study's minimal data set as the underlying data used to reach the conclusions drawn in the manuscript and any additional data required to replicate the reported study findings in their entirety. All PLOS journals require that the minimal data set be made fully available. For more information about our data policy, please see <http://journals.plos.org/plosone/s/data-availability>.

***Data availability***

***Data of the study are protected under the protection of health data regulation set by the French National Commission on Informatics and Liberty (Commission Nationale de l’Informatique et des Libertés, CNIL) in line with the European regulations and the Data Protection Act .***

***The data can be available upon reasonable request to the co-principal investigator of the study (josiane.warszawski@inserm.fr). The French law forbids us to provide free access to EPICOV data; access could however be given by the EPICOV steering committee after legal verification of the use of the data. Please, feel free to come back to us should you have any additional questions.***

Upon re-submitting your revised manuscript, please upload your study’s minimal underlying data set as either Supporting Information files or to a stable, public repository and include the relevant URLs, DOIs, or accession numbers within your revised cover letter. For a list of acceptable repositories, please see <http://journals.plos.org/plosone/s/data-availability#loc-recommended-repositories>. Any potentially identifying patient information must be fully anonymized.

Important: If there are ethical or legal restrictions to sharing your data publicly, please explain these restrictions in detail. Please see our guidelines for more information on what we consider unacceptable restrictions to publicly sharing data: <http://journals.plos.org/plosone/s/data-availability#loc-unacceptable-data-access-restrictions>. Note that it is not acceptable for the authors to be the sole named individuals responsible for ensuring data access.

We will update your Data Availability statement to reflect the information you provide in your cover letter.

4. We note that you have included the phrase “data not shown” in your manuscript. Unfortunately, this does not meet our data sharing requirements. PLOS does not permit references to inaccessible data. We require that authors provide all relevant data within the paper, Supporting Information files, or in an acceptable, public repository. Please add a citation to support this phrase or upload the data that corresponds with these findings to a stable repository (such as Figshare or Dryad) and provide and URLs, DOIs, or accession numbers that may be used to access these data. Or, if the data are not a core part of the research being presented in your study, we ask that you remove the phrase that refers to these data.

***A supporting information file was added, as well as a sentence in the Results section.***

**Women appeared to be more reluctant to vaccination in general than men (OR=1.33 (95% CI: 1.26-1.40)), and even more so with regard to the Covid-19 vaccine (OR=1.88 (95% CI: 1.79-1.97)), although they were more afraid than men of being infected and being seriously ill, than men, and they took a Covid-19 test more often (S1 Fig).**

***In the Discussion session, reference was made to S1 Fig.***

5. We note you have included a table to which you do not refer in the text of your manuscript. Please ensure that you refer to Table 3 in your text; if accepted, production will need this reference to link the reader to the Table.

***We included the reference to Table 3 in the following sentence: “The presence of a child was also not equally important according to the type of vaccine: it increased the probability of being hostile to Covid-19 vaccine (OR=0.12 (95% CI: 1.06-1.18) but was not significant for vaccination in general (Table 3)”***

6. Please review your reference list to ensure that it is complete and correct. If you have cited papers that have been retracted, please include the rationale for doing so in the manuscript text, or remove these references and replace them with relevant current references. Any changes to the reference list should be mentioned in the rebuttal letter that accompanies your revised manuscript. If you need to cite a retracted article, indicate the article’s retracted status in the References list and also include a citation and full reference for the retraction notice.

***Following Reviewer #1’s remarks on references cited in the manuscript, a couple of references were removed due to the publication date, and some were replaced by more current research.***

- ***We removed the following:***

1. [***Bertrand A, Torny D. Libertés individuelles et santé collective : Une étude socio-historique de l’obligation vaccinale. Convention CNRS/ DGS SD5C 03-673: CERMES CNRS UMR 8559 – INSERM U502 – EHESS; 2004 p. 109.***](https://www.zotero.org/google-docs/?0Z9qXR)

[***32.	Boltanski L. Les usages sociaux du corps*. Annales Histoire, Sciences Sociales. 1971;26: 205–233. doi:10.3406/ahess.1971.422470***](https://www.zotero.org/google-docs/?0Z9qXR)

[***37.	Gamble VN. Under the shadow of Tuskegee: African Americans and health care. Am J Public Health. 1997;87: 1773–1778.***](https://www.zotero.org/google-docs/?0Z9qXR)

[***38.	Papon S, Robert-Bobée I. Une hausse des décès deux fois plus forte pour les personnes nées à l’étranger que pour celles nées en France en mars-avril 2020. Insee Focus. 2020 [cited 16 Apr 2021]. Available: https://www.insee.fr/fr/statistiques/4627049***](https://www.zotero.org/google-docs/?0Z9qXR)

[***39.	Salmon DA, Moulton LH, Omer SB, DeHart MP, Stokley S, Halsey NA. Factors associated with refusal of childhood vaccines among parents of school-aged children: a case-control study. Arch Pediatr Adolesc Med. 2005;159: 470–476. doi:10.1001/archpedi.159.5.470***](https://www.zotero.org/google-docs/?0Z9qXR)

[***40.	Marlow LAV, Waller J, Wardle J. Parental attitudes to pre-pubertal HPV vaccination. Vaccine. 2007;25: 1945–1952. doi:10.1016/j.vaccine.2007.01.059***](https://www.zotero.org/google-docs/?0Z9qXR)

[***28.	Cayouette-Remblière J, Lambert A. L’explosion des inégalités. Classes, genre et générations face à la crise sanitaire. L’aube. 2021***](https://www.zotero.org/google-docs/?0Z9qXR)

- ***We replaced the following:***

[***21.	Shim JK. Cultural health capital: A theoretical approach to understanding health care interactions and the dynamics of unequal treatment. J Health Soc Behav. 2010;51: 1–15. doi:10.1177/0022146509361185***](https://www.zotero.org/google-docs/?0Z9qXR)

***(replaced by***

***21. Dubbin LA, Chang JS, Shim JK. Cultural health capital and the interactional dynamics of patient-centered care. Soc Sci Med. 2013;93: 10.1016/j.socscimed.2013.06.014. doi:***[***10.1016/j.socscimed.2013.06.014***](https://doi.org/10.1016/j.socscimed.2013.06.014)***)***

[***27.	Benin AL, Wisler-Scher DJ, Colson E, Shapiro ED, Holmboe ES. Qualitative Analysis of Mothers’ Decision-Making About Vaccines for Infants: The Importance of Trust. Pediatrics. 2006;117: 1532–1541. doi:10.1542/peds.2005-1728***](https://www.zotero.org/google-docs/?0Z9qXR)

***(replaced by***

***27. Dubé E, Vivion M, MacDonald N. Vaccine hesitancy, vaccine refusal and the anti-vaccine movement: influence, impact and implications. Expert Rev Vaccines. 2014;14: 99–117. doi:***[***10.1586/14760584.2015.964212***](https://doi.org/10.1586/14760584.2015.964212)***)***

[***30.	Greenberg MR, Schneider DF. Gender differences in risk perception: effects differ in stressed vs. non-stressed environments. Risk Anal. 1995;15: 503–511. doi:10.1111/j.1539-6924.1995.tb00343.x***](https://www.zotero.org/google-docs/?0Z9qXR)

***(replaced by***

***30. Maxfield S, Shapiro M, Gupta V, Hass S. Gender and risk: women, risk taking and risk aversion. Gender in Management: An International Journal. 2010;25: 586–604. doi:***[***10.1108/17542411011081383***](https://doi.org/10.1108/17542411011081383)***)***

- ***In-text citations were changed as such:***

***Page 11, reference in the following “[...] and on the other by discrimination and mistreatment to which these populations may have been exposed when resorting to the public health system” was changed from***

[***37.	Gamble VN. Under the shadow of Tuskegee: African Americans and health care. Am J Public Health. 1997;87: 1773–1778.***](https://www.zotero.org/google-docs/?0Z9qXR)

***to***

[***35.	Kazemian S, Fuller S, Algara C. The role of race and scientific trust on support for COVID-19 social distancing measures in the United States. PLOS ONE. 2021;16: e0254127. doi:10.1371/journal.pone.0254127***](https://www.zotero.org/google-docs/?broken=TVrx9Q)

***Page 12, reference in the following sentence “These results confirmed a link between vaccine adherence and trust in government, demonstrated prior to the Covid-19 pandemic” was changed from***

[***38.	Papon S, Robert-Bobée I. Une hausse des décès deux fois plus forte pour les personnes nées à l’étranger que pour celles nées en France en mars-avril 2020. Insee Focus. 2020 [cited 16 Apr 2021]. Available: https://www.insee.fr/fr/statistiques/4627049***](https://www.zotero.org/google-docs/?0Z9qXR)

***to***

[***Ward JK, Alleaume C, Peretti-Watel P, Peretti-Watel P, Seror V, Cortaredona S, et al. The French public’s attitudes to a future COVID-19 vaccine: The politicization of a public health issue. Social Science & Medicine. 2020;265: 113414. doi:10.1016/j.socscimed.2020.113414***](https://www.zotero.org/google-docs/?broken=Q7jvAc)

- We added the following reference

***Page 13, reference in the following sentence “In the course of November 2020, the main announcement from officials was made from the French President on November 24th, to announce that the vaccination campaign would be “swift” and “massive”, but that getting vaccinated would not be compulsory” was added***

***France 24. “We must do everything to avoid a third wave and lockdown,” says Macron. France 24. 24 Nov 2020. Available: https://www.france24.com/en/europe/20201124-live-macron-addresses-the-nation-on-path-out-of-lockdown. Accessed 6 Dec 2021.***

Reviewer's Responses to Questions

Reviewer #1: The manuscript is well written, simple, structured language used, and properly organized. Other than some minor problem mentioned in the comments, this research article is quite interesting and easy to understand.

1. In Page 5, Since the cohort procedure is published in another publication, the phrase "Since the cohort protocol is detailed in another publication," requires a bit more background information.

***Additional information on the cohort protocol was added in the manuscript, namely on how estimators representative of the French population were produced.***

***Underlined elements were added to the cohort description:***

**~~Since the cohort protocol is detailed in another publication~~** [**~~[11]~~**](https://www.zotero.org/google-docs/?zxe2pq)**~~, only the essential characteristics will be presented.~~ A stratified random sample of 135,000 people aged 15 and over, was drawn from the tax database of the National Institute of Statistics and Economic Studies (INSEE), which covers 96% of the population living in France but excludes people living in institutional settings, participated in a first wave of the study in May 2020. People belonging to the lowest decile of income were over-represented. A total of 134,391 respondents participated in the first wave of the study (May 2020). A second wave was conducted in November 2020, including questions on attitudes toward vaccination. Respondents who took part in the first wave of the study were invited to take part in this second wave. In all, 107,808 respondents participated in this second wave (81.7% of the respondents of the first wave of the study). Individuals were invited to answer the questionnaire online, or by phone for those who did not have Internet access. Furthermore, a random sample of 10% of people with Internet access was interviewed by phone in order to take into account a method collection effect. The results published in the study have been adjusted by applying the weights established by the National Institute of Statistics and Economic Studies (INSEE) and marginal recalibration in the survey and sampling design to correct for non-participation, so as to produce estimators that are representative of the population. More information about the cohort can be found in another publication** [**[11]**](https://www.zotero.org/google-docs/?nKvEO4)**.**

2. In page 6, It would be better to include the number of people who tested positive for Covid-19 (N=4,036) and whose intention to be vaccinated could be influenced by this fact in the statement "We chose not to include people who tested positive for Covid-19 (N=4,036) and whose intention to be vaccinated could be influenced by this fact."

***After thoughtful consideration, we decided not to include the number of people who tested positive for Covid-19 before the survey. Back in November 2020, the message delivered by the French authorities and later confirmed by instructions given to vaccination centers (***[***https://www.rfcrpv.fr/wp-content/uploads/2021/03/N%C2%B0-118-Novembre-2020-f%C3%A9vrier-2021.pdf***](https://www.rfcrpv.fr/wp-content/uploads/2021/03/N%C2%B0-118-Novembre-2020-f%C3%A9vrier-2021.pdf)***), was that individuals who had tested positive for Covid-19 a couple of months before, could have to wait a couple of months to be vaccinated. Therefore, as the aim of this article was to study factors associated with reluctance to getting vaccinated, we decided not to include those who had tested positive for Covid-19 in this article, as it is too specific a scenario to be considered with the rest of the population. However, it could be interesting to study, in another article, the attitude of this population on vaccination issues.***

3. On Page 10, insert a square bracket"]" in the statement "The third hypothesis involved a gendered relationship to health and environmental risks, which is also the product of primary socializations [29."

***A square bracket was inserted.***

4. Modify and correct the phrase structure of the statement "The older people were, the less they refused vaccination" on page 11.

***The sentence was replaced with “As they got older, respondents were less likely to refuse vaccination.”***

5. In page 13, Instead of giving a direct link, such as "“(<https://www.santepubliquefrance.fr/etudes-et-enquetes/coviprev-une-enquete-pour> suivre-l-evolution-des-comportements-et-de-la-sante-mentale-pendant-l-epidemie-de covid- 19#block-249162)” it would be better to make it a reference and cite in.

***The reference was added and cited in the reference list.***

6. Few references in the text are from 2004 and 2010, and efforts should be made to provide relevant recent references that are no more than five years old.

***The reference list was changed according to this comment. References from 2004 and 2010 were removed/replaced by a more current reference.***

Reviewer #2: In the current research article entitled "The social specificities of hostility toward vaccination against Covid-19 in France", by Bajos et al., have studied/surveyed opposition toward vaccination against Covid-19 in France. They found that, in France a gendered reluctance toward vaccination in general but even more so regarding vaccination against COVID-19. Further, not only people at the bottom of the social hierarchy but also immigrants and descendants of immigrants were all more reluctant to the Covid-19 vaccine. This article addresses a research topic of great interest, which is under intense investigation in the past 2 years and the manuscript is generally well-written. However, this reviewer has certain suggestions that would help produce a more comprehensive overview of the topic:

Suggestions:

1. At least one additional Figure (illustration) may be provided as to highlight the summary or prospect of this study.

***A flowchart of the national EpiCoV cohort was added (Fig 1) following the Sample information section, so as to provide better understanding of the study design.***

2. One paragraph can be added to discussion the Government of France efforts to improve COVID-19 vaccination during the survey period.

***The following paragraph has been added in the discussion:***

**“It should be added that during the period of the survey, the vaccination campaign was still being developed by French authorities. In the course of November 2020, the main announcement from officials was made from the French President on November 24th, to announce that the vaccination campaign would be “swift” and “massive”, but that getting vaccinated would not be compulsory** [**[40]**](https://www.zotero.org/google-docs/?3cPBdy)**. It would be coherent to assume that the announcement did not influence the attitude towards the Covid-19 vaccine, and that the link between confidence in the government and reluctance to getting vaccinated was developed before the survey period.”**
